# Dominant TOM1 mutation associated with combined immunodeficiency and autoimmune disease

**DOI:** 10.1038/s41525-019-0088-5

**Published:** 2019-06-27

**Authors:** Salla Keskitalo, Emma M. Haapaniemi, Virpi Glumoff, Xiaonan Liu, Ville Lehtinen, Christopher Fogarty, Hanna Rajala, Samuel C. Chiang, Satu Mustjoki, Panu Kovanen, Jouko Lohi, Yenan T. Bryceson, Mikko Seppänen, Juha Kere, Kaarina Heiskanen, Markku Varjosalo

**Affiliations:** 1 Institute of Biotechnology, University of Helsinki, Helsinki, Finland; 2 Centre for Molecular Medicine Norway, University of Oslo, Oslo, Norway; 3 Center for Hematology and Regenerative Medicine, Karolinska Institutet, Stockholm, Sweden; 4 Biomedicum Stem Cell Center, University of Helsinki, Helsinki, Finland; 5 Research Unit of Biomedicine, Medical Microbiology and Immunology, University of Oulu, Oulu, Finland; 6 Päijät-Häme Central Hospital, Lahti, Finland; 7 Folkhälsan Institute of Genetics, Helsinki, Finland; 8 Abdominal Center Nephrology, University of Helsinki and Helsinki University Hospital, Helsinki, Finland; 9 Research Programs Unit, Diabetes, and Obesity, University of Helsinki, Helsinki, Finland; 10 Hematology Research Unit Helsinki, Helsinki University Hospital Comprehensive Cancer Center, Helsinki, Finland; 11 Department of Clinical Chemistry, University of Helsinki, Helsinki, Finland; 12 Center for Hematology and Regenerative Medicine, Karolinska Institutet, Huddinge, Sweden; 13 Department of Pathology, University of Helsinki and Helsinki University Hospital, Helsinki, Finland; 14 Rare Disease Center, Hospital for Children and Adolescents and Adult Immunodeficiency Unit, Inflammation Center, University of Helsinki and Helsinki University Hospital, Helsinki, Finland; 15 Department of Biosciences and Nutrition, Karolinska Institutet, Stockholm, Sweden; 16 Research Programs Unit, Molecular Neurology, University of Helsinki, Helsinki, Finland; 17 School of Basic and Medical Biosciences, King’s College London, Guy’s Hospital, London, UK; 18 Hospital for Children and Adolescents, University of Helsinki and Helsinki University Hospital, Helsinki, Finland

**Keywords:** Diseases, Immunological disorders

## Abstract

Mutations in several proteins functioning as endolysosomal components cause monogenic autoimmune diseases, of which pathogenesis is linked to increased endoplasmic reticulum stress, inefficient autophagy, and defective recycling of immune receptors. We report here a heterozygous *TOM1* p.G307D missense mutation, detected by whole-exome sequencing, in two related patients presenting with early-onset autoimmunity, antibody deficiency, and features of combined immunodeficiency. The index patient suffered from recurrent respiratory tract infections and oligoarthritis since early teens, and later developed persistent low-copy EBV-viremia, as well as an antibody deficiency. Her infant son developed hypogammaglobulinemia, autoimmune enteropathy, interstitial lung disease, profound growth failure, and treatment-resistant psoriasis vulgaris. Consistent with previous knowledge on TOM1 protein function, we detected impaired autophagy and enhanced susceptibility to apoptosis in patient-derived cells. In addition, we noted diminished STAT and ERK1/2 signaling in patient fibroblasts, as well as poor IFN-γ and IL-17 secretion in T cells. The mutant TOM1 failed to interact with TOLLIP, a protein required for IL-1 recycling, PAMP signaling and autophagosome maturation, further strengthening the link between the candidate mutation and patient pathophysiology. In sum, we report here an identification of a novel gene, *TOM1*, associating with early-onset autoimmunity, antibody deficiency, and features of combined immunodeficiency. Other patient cases from unrelated families are needed to firmly establish a causal relationship between the genotype and the phenotype.

## Introduction

Primary immunodeficiencies that present with prominent autoimmunity provide insights into the molecular mechanisms of tolerance breakdown. The common defective genes in these conditions encode proteins that affect T-cell development and function. Examples include loss of FOXP3 that governs the regulatory T-cell development, and gain or loss of the STAT proteins that polarize T cells to different helper subsets.^1–8^ In addition, defects in major immunological signaling molecules and proteins that govern the actin cytoskeleton formation can lead to T-cell driven autoimmunity.^2,7^

Several proteins functioning in the endolysosomal system can also cause monogenic autoimmune diseases when defective^9–11^ (online Supplementary Information Table S1). The pathogenesis is linked to increased endoplasmic reticulum stress, inefficient autophagy, and impaired recycling of immune receptors. Herein, we report a previously undescribed, dominantly inherited immune disease in two related patients that carry a missense mutation in *TOM1* (*Target of Myb protein 1*). TOM1 is an adaptor protein needed for the maturation of autophagosomes and their fusion with lysosomes.^12^ TOM1 also inhibits Toll-like receptor (TLR) signaling and participates in immune receptor recycling.^13,14^ Clinically, the index patient presents with a relatively mild disease, whereas in the child the condition is aggressive and fatal. This phenotypic heterogeneity is common in monogenic immune diseases and points to additional genetic modifiers in disease presentation.

## Results

We evaluated a mother–son pair presenting with childhood-onset autoimmune disease and combined immunodeficiency (Table 1, and detailed case descriptions in Supplementary Information). The index case (Patient 1, II.2) suffered from recurrent respiratory tract infections and joint pain since her early teens, and was diagnosed with seronegative oligoarthritis and hypogammaglobulinemia at age 16 (plasma IgG of 3,0 g/l, IgA 0,15 g/l, and IgM 0,17 g/l). Intravenous immunoglobulin replacement was initiated but soon discontinued due to adverse effects. In her early 30 s, she developed chronic diarrhea with normal endoscopic findings and was noted to have persistent low-copy EBV viremia, between 200–800 viral copies/ml. She is currently receiving peroral prednisolone, methotrexate, and subcutaneous immunoglobulin replacement.

**Table 1:** Clinical and immunological features of the study participants

	Patient 1, II.2	Patient 2, III.1
Sex	Female	Male
Current age	32	9
Growth	Normal growth	Growth failure (–4.5 SD)
*Infection susceptibility*		
	Respiratory tract infections	Respiratory tract infections
	Low-copy EBV viremia (100–2600 copies/ml)	
*Immune dysregulation*		
	Atopic eczema	Psoriasis
	Seronegative polyarthritis	Lymphocytic interstitial pneumonitis
	Chronic diarrhea	Autoimmune enteropathy
*Laboratory features* ^*a*^		
Lymphocytes (1300–3600)	660 ↓	1670
B cells (CD19 + ) (100–500)	20 ↓	320
Switched memory B cells (CD27 + IgD−IgM−) (6.5%–29.2%)	0 ↓	0 ↓
T cells (CD3 + CD4 + ) (300–1400)	491	722
T cells (CD3 + CD8 + ) (200–1200)	170 ↓	300
T_reg_ (FOXP3 + CD25 + ) (2.8%–6.4%)	5.8	5.5
T_reg_ suppressive capacity	Normal	Reduced
NK cells (CD3-CD16 + 56 + ) (90–600)	40 ↓	20 ↓
Plasmacytoid dendritic cells (lin-HLA-DR + CD123 + CD11c–)	0.04 ↓	0.04 ↓
Monocytoid dendritic cells (lin-HLA-DR + CD13–CD11c + )	0.04 ↓	0.04 ↓
*Immunoglobulins*		
IgG (6.8-15.0 g/l)	0.9 ↓	7.5 (substitution)
IgA (0.52-4.02 g/l)	0 ↓	< 0.1 ↓
IgM (0.47-2.84 g/l)	0.07 ↓	0.1 ↓
IgE (0-110 IU/l)	< 4	5 kU/l

Child’s deviant values are indicated according to pediatric references

^a^See Online Supplementary Information Tables S2 and 3 for more comprehensive workup

Her son (Patient 2, III.1) was evaluated for failure to thrive at age 6 months and was subsequently diagnosed with autoimmune enteropathy and lung disease that later evolved to lymphocytic interstitial pneumonitis (LIP). At age 3, he developed eczema that, by age 6, had become generalized and treatment resistant, resembling psoriasis vulgaris (Supplementary Fig. S1). He displayed profound growth failure (–4.5 SD) and hypogammaglobulinemia since age 12 months. During the course of the study, he received peroral tacrolimus, methotrexate and subcutaneous immunoglobulin replacement, and recently underwent allogeneic stem cell transplant, with a temporary resolution of autoimmune symptoms. Within 6 months, however, he rejected the allograft and the disease returned. He died one year after the transplant for progressive pulmonary fibrosis.

Detailed immunological workup is presented in online Supplementary Information Tables S2 and S3. Both patients had low numbers of dendritic cells, NK cells and switched memory B cells; additionally, the regulatory T-cell function was impaired in the severely affected son (Table 1 and Tables S2 and S3). The T-cell compartment showed increased naive as well as decreased T-cell effector memory (TEM) and effector memory RA (TEMRA) subsets, suggesting impaired T-cell maturation. Both patients had poor IFN-γ and IL-17 secretion upon stimulation, even though enhanced Th17 response is considered a hallmark of T-cell-mediated autoimmune disease.^6^

**Fig. 1:**
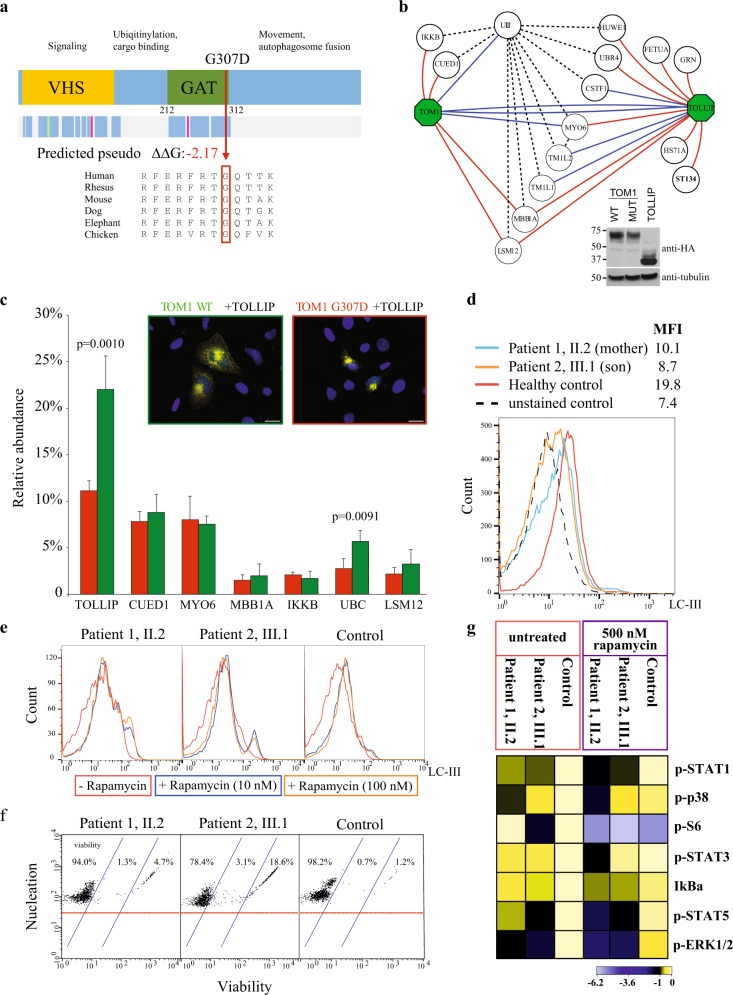
**a** Schematic representation of *TOM1* and the observed mutation in the GAT (GGA and Tom1) domain. Mutated G307 residue and its evolutionary conservation is shown together with predicted reduced stability (predicted pseudo ΔΔ*G*). **b** The AP-MS analysis of TOM1 wild type (WT), G307D mutant and TOLLIP WT confirmed reciprocally the known interactions of TOM1 and TOLLIP (blue edges) as well as identified several novel (red) interactions. The black dashed line indicates known prey-to-prey interactions. Inset: expression of all the constructs was confirmed with western blotting using anti-HA antibody. Tubulin was used as a loading control. Blots derive from the same experiment and they were processed in parallel. **c** The quantitative interactome analysis of TOM1 WT (green) and G307D mutant (red) showed significantly decreased binding of the G307D with polyubiquitin-C (UBC) and TOLLIP compared to the WT. Error bars indicate standard deviation. The *p*-values were calculated using Student’s *t*-test. Inset: TOLLIP efficiently recruits TOM1 WT (left) to early endosomes while recruitment of G307D (right) is hampered. HeLa cells were co-transfected with Myc-tagged WT or G307D TOM1 (green and red rectangle, respectively) and HA-tagged TOLLIP. Merged images are shown, where scale bar represents 20 μm. **d** Diminished LC3 staining of patient PBMCs indicates decreased autophagy. Son (III.1) is indicated with orange, mother (II.2) with blue, and control with red solid line. Interestingly, ~50% of son’s lymphocytes overlapped with the unstained control population (black dashed line). MFI, mean fluorescence intensity. **e** Rapamycin fails to induce autophagy in PBMCs. Line colors indicate rapamycin concentrations. **f** PBMCs show elevated numbers of apoptotic and dead cells in both patients. **g** Intracellular signaling network responses in patient skin fibroblasts reveals alterations of key pathway components phosphorylation levels at basal state and after rapamycin treatment. Heat map shows the calculated Log2 ratios of medians of panel/channel values relative to untreated control. Analyzed phosphoepitopes were STAT1 (anti-pTyr701), p38 (anti-pThr180/pTyr192), S6 (anti-pSer235/pSer236), STAT3 (anti-pTyr705), STAT5 (anti-pTyr694), and ERK1/2 (anti-pThr202/pTyr204)

As the clinical presentation pointed to dominantly inherited immunodeficiency, we performed trio-whole-exome sequencing of the mother, the only child and his healthy father. We filtered the data for dominant coding sequence variants shared by the affected individuals and not present in the public and in-house control datasets. This resulted in 13 variants, of which three were damaging to the protein structure and affected a conserved residue (online Supplementary Information Table S4). A variant in Target of Myb protein 1 -gene (*TOM1*; chr22: 35728994 G > A, p.G307D, Fig. 1a) was considered the most likely candidate, as it was absent from control datasets and deleteriously affected a conserved residue (SIFT prediction “deleterious”, PolyPhen prediction “probably damaging”). TOM1 is highly expressed in immune cells and participates in autophagy, ubiquitination, and receptor recycling; pathways, which are implicated in several monogenic autoimmune conditions (refs ^10,11^ and Table S1).

The expression of WT and mutant TOM1 were identical (Fig. 1b and Supplementary Fig. S1B); therefore, we hypothesized that the mutation affects cellular protein–protein interactions of TOM1, as is the case in many pathogenic missense variants that do not lead to absent protein expression.^15^ Therefore, we used Flp-In T-REx 293 cell lines that ectopically expressed mutant or wild type (WT) streptavidin-tagged TOM1 and analyzed the interactions by affinity-purification mass spectrometry (AP-MS). The AP-MS analysis confirmed the known and identified several novel interactions (Fig. 1b). In the quantitative interactome analysis the mutant TOM1 had statistically significant decreased interactions with ubiquitin C (2.8% mutant, 5.7% WT) and TOLLIP (11.2% mutant, 22.0% WT) (Fig. 1c). TOLLIP is an endosomal protein that negatively regulates TLR (toll-like receptor) and innate immune signaling, and is associated with various autoimmune conditions.^16^ Normally upon transient co-expression TOLLIP recruits TOM1 to endosomes,^13^ however this function was impaired with the mutant TOM1 (Fig. 1c), although WT and mutant TOM1 showed similar cytosolic localization (Supplementary Fig. S2). These data suggest that the mutation disrupts the TOM1–TOLLIP complex formation, which then hampers the packaging of ubiqitinylated proteins to endosomes.

To further understand how TOM1–TOLLIP complex functions in endosomal cargo delivery, we performed BioID experiments with stable Flp-In T-REx 293 cells lines. The BioID method involves bait-fused modified biotin ligase (BirA) biotinylating close-proximity proteins allowing identification of transient and proximal interactions.^17^ In this analysis TOM1 and TOLLIP localized to endo/exosomal vesicles and had multiple common interaction partners. As defects in the vesicular trafficking protein LRBA cause monogenic autoimmune disease similar to our patients,^18^ we even mapped the interactions of this protein. LRBA interacted with both TOM1 and TOLLIP but had molecular context mainly in the Golgi/trans-Golgi transport. This suggests that while TOM1 localizes to vesicles, LRBA functions mainly in the Golgi-endosomal axis, and the molecular pathogenesis of TOM1 and LRBA deficiencies are distinct from each other (Supplementary Fig. S2).

Ubiqitinylated proteins are degraded by the endolysosomal system. The process is tightly connected to autophagy, in which the cell digests its own organelles to create energy and nutrients. To evaluate whether autophagy was disturbed in patient lymphocytes, we analyzed the expression of LC3—which indicates the presence of autophagosomes—after serum starvation (Fig. 1d). Lymphocytes from both patients showed decreased LC3 staining, indicating a low autophagosome count compared to a healthy control. Rapamycin and other rapalogs are potent inducers of autophagy, and in an attempt to control autoimmunity we treated patient 2 with everolimus. Contrary to our expectations, this led to flares of eczema and respiratory distress, symptoms that quickly stabilized once the drug was stopped. In vitro, rapamycin treatment on patient lymphocytes failed to increase the number of autophagosomes (Fig. 1e). These observations point to impaired autophagy, most probably due to defects in autophagosome formation.

As impaired autophagy often leads to increased apoptosis,^19^ we also evaluated the proportion of apoptotic and dead PBMCs in the patients, which were increased compared to healthy control (Fig. 1f). Similar phenotypes of increased apoptosis and defective autophagy have been reported in common autoimmune conditions such as systemic lupus erythematosus and in the monogenic autoimmune disease caused by LRBA-deficiency.^18,20^ Impaired autophagy is also linked to increased endoplasmic reticulum stress;^21^ however, we did not detect evidence of abnormal ER stress response when staining inflamed gut and respiratory mucosa paraffin sections with BiP antibody.

Besides autophagy, TOM1 participates in receptor recycling.^12,13^ We therefore examined the patient lymphocytes for their ability to express CTLA4, IL-1 and IL-6 receptors, as increased IL-6 signaling and impaired CTLA-4 expression give monogenic autoimmune phenotypes similar to our patients.^6,22^ All tests were normal (Supplementary Fig. S2). To further understand the effect of the mutation on major immune signaling pathways, we measured activating phosphorylation of components of Jak-STAT, NF-κB, and Ras-MAPK signaling pathways in patient skin fibroblasts (Fig. 1g). Interestingly, ERK1/2 phosphorylation was significantly downregulated in both patients (log2 fold change > 1, Fig. 1g) indicating dysfunctional MAPK signaling. Both patients also showed impaired STAT1 and STAT5 tyrosine phosphorylation that further diminished upon rapamycin treatment. Single-gene defects in both of these STATs lead to early-onset autoimmunity by disrupting T-cell development and polarization.^1,4,5,8^ These results suggest that the TOM1 mutation affects MAPK and JAK-STAT signaling. This most likely explains the observed Th1 and Th17 cell developmental defects (Table S3) and contributes to poor regulatory T-cell function.

## Discussion

We associate heterozygous *TOM1* mutation to a combined immunodeficiency and early-onset autoimmunity. The *TOM1* mutation disrupts the TOM1–TOLLIP complex formation and the patient cells show defects in autophagy, susceptibility to apoptosis, and downregulation of multiple key signaling effectors, including ERK1/2, STAT1, and STAT5. The difference in disease severity suggest that additional genetic and environmental factors modify the phenotype. As in all ultra-rare diseases initially reported in single families, additional cases from unrelated pedigrees are needed to determine with complete certainty a causal relationship between mutations in TOM1 and the clinical phenotype described here, and to better understand the molecular pathogenesis of *TOM1* mutations.^23^

## Methods

### Study participants

The study was conducted in accordance to the principles of the Helsinki Declaration and was approved by the Helsinki University Hospital Ethics Committee. Written informed consent was obtained from the mother and father, as well as their permission for the child. Also all other participants signed written informed consent. The authors affirm that human research participants provided informed consent, for publication of the images in the Supplementary Fig. S1.

### DNA extraction and whole-exome sequencing, and validation of the candidate mutations

Genomic DNA was extracted from EDTA blood samples using Qiagen FlexiGene DNA kit (Qiagen). Libraries were processed according to Agilent SureSelect Target Enrichment System (Agilent Technologies) for Illumina Paired-End Sequencing Library (Illumina) using SureSelect Human All Exon V5 capture library (Agilent Technologies). Libraries were sequenced with 101 bp read length (HiSeq1500 sequencing platform, Illumina), with ~120x depth.

The read mapping, variant calling, and genome annotation were performed as described previously.^3^ We first analyzed the parent and child exomes separately. After variant calling, we filtered the data for coding mutations with minor allele frequency of < 0.01 in control databases (The Exome Aggregation Consortium (ExAC), 1000 Genomes, NHLBI Exome variant server and UK TWIN and ALSPAC study cohorts (2–4), as well as in-house databases. We then focused the search on rare, damaging variants in known Mendelian disease-causing genes; however, neither patient did have damaging variants with appropriate phenotype and heritage model.

We next targeted the analysis on shared variants between the affected individuals. We filtered the data for shared coding mutations not present in control databases (The Exome Aggregation Consortium (ExAC), 1000 Genomes, NHLBI Exome variant server and UK TWIN and ALSPAC study cohorts (2–4), as well as in-house databases) (see Table S4). This analysis recovered two functionally plausible candidate variants, one in TOM1 and another in TBC1D31.

As the phenotype in the child was more severe than in the parent, we also analyzed the data for damaging, coding variants that were heterozygous in the mother, and homozygous or compound heterozygous in the parent. No plausible variants were found.

The candidate mutations were verified by sequencing from fibroblast DNA and RNA/cDNA (Eurofins Genomics Germany GmbH, Germany) (Fig. S1B). The mutant allele was expressed similarly to WT. Primers used for sequencing (GAGGAGCTGCTCATCGTCAATG), forward primer for gDNA and cDNA PCR (TOM1-forward1 CTGGAGCTCATCCCTCAGAT), reverse primer for cDNA PCR (TOM1-reverse1 TACCTCTTTCCGTTGGTCAGC) and reverse primer for gDNA PCR (TOM1-reverse2 AGCTGGGATGAGAGGTTGC).

### B- and T-cell immunophenotyping

Fresh EDTA blood samples or PBMCs were used for B and T lymphocyte immunophenotyping. Four or 6-color flow cytometry panel with mAbs against the surface antigens IgM, IgD, CD3, CD4, CD8, CD16⁄56, CD19, CD21, CD27, CD33, CD34, CD38, CD45, CD56, CD57, CD133, HLA-DR, CD62L, CD45RA, and CD45RO (BD Biosciences) were applied.^24^ The memory status of T cells was studied with an antibody panel, including anti-CD45, -CD3, -CD4, -CD45RA, and -CCR7 (R&D Systems).^3,24^

Evaluation of T-cell responses is described in detail elsewhere.^24^ For the assessment of T-cell activation, fresh mononuclear cells were stimulated for 6 h with anti-CD3, anti-CD28, and anti-CD49d (BD Biosciences). The cells were analyzed using a 4- or 6-color flow cytometry panel with mAbs against the antigens CD45, CD3, CD4, CD8, CD16, CD56, CD45, CD45RA, TCR-γ, CCR7, IFN-γ, and tumor necrosis factor (TNF).

### Analysis of T- and NK-cell cytotoxicity

Evaluation of T- and NK-cell responses is described in detail elsewhere.^24,25^ For the assessment of T-cell activation and degranulation, fresh mononuclear cells (MNCs) were stimulated for 6 h with anti-CD3, anti-CD28, and anti-CD49d (BD Biosciences). For NK-cell degranulation, cytokine and cytotoxicity assays, fresh MNCs or FACS-sorted CD3-CD16/56 + NK cells were stimulated with K562 target cells for 6 h. The cells were analyzed using 4- or 6-color flow cytometry panel with mAbs against the antigens CD45, CD3, CD4, CD8, CD16, CD56, CD45, CD45RA, TCRγ, CCR7, IFN-γ and TNF-α. Additionally, standard 4 h chromium 51 (51Cr)-release assays were performed according to established protocols for clinical samples using magnetic bead—separated CD3 + CD8 + T cell or CD3−CD56 + NK cell subsets.^24,25^

### Phenotyping of Th17 and T_reg_ cells

Phenotyping of IL-17-positive Th17 cells and T_regs_ is described in detail elsewhere.^3^ Briefly, fresh PBMCs were stimulated for 16 h with anti-CD3/anti-CD28 beads in the presence of Brefeldin A. Thereafter, the cells were fixed, permeabilized and stained with anti-CD4, -CD69-APC, and IL-17A (BD Biosciences) and analyzed with FACSAria II or FACSCanto II or LSRFortessa flow cytometer.

Th1/Th17 CD4 + memory cells were detected from whole blood by a four-color flow cytometry panel with mAbs (CD45RA-FITC, CD4-PerCP, CXCR3-APC, and CCR6-BV421 (BioLegend, San Diego, CA)) against surface antigens similarly to ref. ^26^ eZKine^TM^ Th1/Th17 Whole Blood Intracellular Cytokine Kit (eBioSciences) was used to measure the INF-gamma production.

T_regs_ were immunophenotyped from fresh blood with surface markers against CD4, CD25, and FOXP3 (BD Biosciences).^3^ For evaluation of T_reg_ suppressor capacity, CD4^+^CD25^+^CD127^-^ T_reg_ cells were sorted from whole blood using Human CD4 + T Cell Enrichment Cocktail (Stemcell Technologies) and fluorescence-activated cell sorting with mAbs against CD4, CD25, and CD127 (BD Biosciences). The cells were incubated for 6 days with CFSE-labeled autologous responder T cells in a ratio of 1:2. Anti-CD3/anti-CD28 beads (Life Technologies) were used as stimulus. CD4^+^ cells were analyzed using FACSAria II flow cytometer (BD Biosciences). The suppression percentage was calculated with the following formula: 100 – [(% proliferation in presence of T_reg_/% proliferation in absence of T_reg_)x100].^3,27^

### Generation of inducible Flp-In™ T-REx 293 cell lines

The cDNA constructs containing WT and p.G307D mutant *TOM1* and LRBA full length coding sequences were ordered as synthetic genes and cloned into pTO_HA_StrepIII_c_GW_FRT,^28^ pTO_MYC_BirA_c,^29^ and pcDNA-DEST40_3xV5^30^ destination vectors. The TOLLIP cDNA was obtained as a gateway compatible entry-clone and cloned into pTO_HA_StrepIII_n_GW_FRT^28^ and pTO_MYC_BirA_n destination vectors. Generation and culture of Flp-In T-REx 293 (ThermoFischerScientific) cell lines was performed as previously described.^28,31^

### Western blotting

For WB analysis, 5 × 105 Flp-In™ T-Rex 293 cells were seeded to six-well plates, induced with tetracycline, harvested to Laemmli Sample Buffer, boiled and ran to sodium dodecyl sulfate polyacrylamide gel electrophoresis (SDS-PAGE) gel. Proteins were transferred onto nitrocellulose membrane and detected with anti-HA (HA-11, Covance), anti-V5 (Invitrogen), or anti-c-Myc primary (9E10, Santa Cruz) and horseradish peroxidase (HRP)-conjugated secondary antibody. Anti-alpha Tubulin antibody (ab7291, Abcam) was used as a loading control. Signal was visualized by chemiluminescence.

### Immunofluorescence

Hela cells were seeded onto coverslips on four-well plates, transiently transfected with Fugene6 (Promega) according to manufacturer’s instructions. The expression of the constructs was induced with tetracycline and after 24 h the Hela cells were fixed with 4% PFA. Cell were stained with anti-HA antibody, anti-V5 antibody and visualized with Alexa-488 goat anti-mouse IgG (Life Technologies, Thermo Fisher Scientific) or Alexa594 goat anti-rabbit IgG. Nuclei were stained with DAPI prior to mounting with Vectashield (Vector Laboratories). Fluorescent microscope, Zeiss Axio Scope.A1 (Zeiss, Oberkochen, Germany) with x40 magnification was used to image the samples. The image files were processed with Zen (Zeiss, Oberkochen, Germany), CorelDRAW X7 and ImageJ softwares.

### Affinity purification, BioID (purification of BirA*), and mass spectrometry

Affinity purification (AP) and BioID experiments together with mass spectrometry were performed as previously described.^29^ Briefly, for AP ~5 × 10^7^ cells were lysed in HNN lysis buffer (50 mM HEPES pH 8.0, 150 mM NaCl, 5 mM EDTA, 0.5% NP-40, 50 mM NaF, 1.5 mM Na3VO4, 1.0 mM PMSF (phenylmethanesulfonylfluoride) and 10 μl/ml protease inhibitor cocktail, Sigma). For BioID ~5 × 10^7^ cells (5 × 15 cm dishes) in three biological replicates were induced with 2 µg/ml doxycycline and 50 μM biotin for 24 h. After induction, cells were washed and harvested under harsher conditions using 0.1% SDS and 80 U/ml Benzonase Nuclease (Santa Cruz Biotechnology) in HNN lysis buffer. The proteins were bound using Strep-Tactin sepharose and Bio-Spin chromatography columns (Bio-Rad) and eluted with D-biotin (Thermo Scientific). After C18-purification samples were analyzed with Orbitrap Elite ETD hybrid mass spectrometer as described in ref. ^29^ Each sample was analyzed in triplicates. The biological clustering and pathway analysis was conducted using DAVID bioinformatics database.^32^

### Small interfering RNA (siRNA) knock-down of TBC1D31

The ON-TARGETplus siRNA SMARTpool targeting human TBD1D31 (GUGAUGAUCUACAACGAAA, UGGCUGAAAUUGUUCGAUA, GCAGAUGCCUAUAGACGAA, GAUAAAUGCGGCUGUAGAA) and the non-targeting pool (UGGUUUACAUGUCGACUAA, UGGUUUACAUGUUGUGUGA, UGGUUUACAUGUUUUCUGA, UGGUUUACAUGUUUUCCUA) (Dharmacon, USA) were transfected to parental HAP1 cells (Horizon Discovery, UK) with DharmaFECT according to manufacturer’s protocols using recommended 100 nM final siRNA concentration. Twenty-four hour post transfection, the transfection medium was replaced with normal complete medium. Cells were analyzed for autophagy induction 48 h after transfection using FlowCellect™ Autophagy LC3 Antibody-based Assay Kit (Merck Millipore). For autophagy induction, the cells were either starved for 3 h (induced) or left untreated (control).

### Immunohistochemical staining of GRP78/BiP

Immunohistochemisty (IHC) of lung and duodenal biopsy paraffin sections was performed according to standard techniques using GRP78/BiP mAb (ab21685, Abcam). Biopsy slides from three healthy individuals (C1, C2, C3) were used as controls.

### CTLA4 staining of peripheral blood lymphocytes

Mononuclear cells purified from heparinized blood of the two patients and two healthy controls (C4, C5) were stimulated either with CD3/CD28 Dynabeads (Invitrogen) and IL-2 (Proleukin), phytohemagglutinin and Ionomycin, or growth media alone, for 5 days. Cells were then evaluated for CTLA4 (Clone BNI3) expression in CD4^+^ T cells by flow cytometry.

### LC3 staining of peripheral blood mononuclear cells

Fresh PBMCs from the patients and three healthy controls (C6, C7, C8) were incubated overnight in RPMI supplemented with 10% FCS and 1% Penicillin–Streptomycin. To analyze the LC3 expression by FACS, the next day cells were either serum-starved or let grow for 6 h. The LC3 staining was done according to FlowCellect™ Autophagy LC3 Antibody-based Assay Kit (Merck Millipore). The LC3 expression was measured according to kit instructions using Guava easyCyte 6-2 L (Merck Millipore). All samples were analyzed in triplicate.

### Apoptosis assay

Fresh PBMCs from the patients and three healthy controls (C6, C7, C8) were stained according to ViaCount® Reagent (Merck Millipore) instructions immediately after isolation. The amount of viable, apoptotic and dead cells was measured according to instructions using Guava easyCyte 6-2 L (Merck Millipore).

### Phospho-flow analysis by mass cytometry

We performed skin biopsy to establish fibroblast cell lines from both patients and a healthy control (C9). Once established, the cells were incubated for 16 h with 500 nM rapamycin or left untreated. The following day cells were fixed with paraformaldehyde, permeabilized with cold methanol, and stained with Maxpar Signaling I Panel Kit according to Maxpar Phoshophoprotein Staining Protocol provided by manufacturer (Fluidigm). Data was collected on a CyTOF mass cytometer (Fluidigm). For data analysis we utilized Cytobank premium (cytobank.org).

### Reporting summary

Further information on experimental design is available in the Nature Research Reporting Summary linked to this paper.

## Supplementary information

Supplementary material

Supplementary Data S1

Supplementary Data S2

Reporting Summary

## Data Availability

The raw data that support the findings of this study are available from the corresponding author upon reasonable request. The sequencing data from the two study patients are available through the Institute for Molecular Medicine Finland (FIMM) Data Access Committee (DAC) for authorized researchers who have IRB/ethics approval and an institutionally approved study plan. For more details, please contact the FIMM DAC (http://fimm-dac@helsinki.fi).

## Supplementary information

**Supplementary Information** accompanies the paper on the *npj Genomic Medicine* website (10.1038/s41525-019-0088-5).
