# Supplementary material for: Dominant TOM1 mutation associated with combined immunodeficiency and autoimmune disease
Source: NPJ Genom Med. 2019 Jun 27;4:14. doi: 10.1038/s41525-019-0088-5 (PMC6597545; doi:10.1038/s41525-019-0088-5)
Supplement: Supplementary file 1 — Supplementary material [file 41525_2019_88_MOESM1_ESM.pdf]

Supplementary Information

Supplement to: “Dominant TOM1 mutation associated with combined immunodeficiency and autoimmune disease” Salla Keskitalo*, Emma M. Haapaniemi*, Virpi Glumoff, Xiaonan Liu, Ville Lehtinen, Christopher Fogarty, Hanna Rajala, Samuel C. Chiang, Satu Mustjoki, Panu Kovanen, Jouko Lohi, Yenan T. Bryceson, Mikko Seppänen, Juha Kere, Kaarina Heiskanen, and Markku Varjosalo.

Table of contents

Case descriptions

Table S1. Genes affecting vesicular transport and causing monogenic autoimmune disease.

Figure S1. Family pedigree, sequencing of TOM1 mutation in primary patient fibroblast, clinical manifestations in patient 2.

Table S2. Immunological features of study participants.

Table S3. Lymphocyte differentials of study participants.

Table S4. List of shared coding variants between the patients.

Figure S2. Differential molecular context of TOM1 and LRBA and role in CTLA4 regulation.

Figure S3. LC-III autophagy in TOM1 knock-out and TBC1D31 knock-down (siRNA) cells.

Figure S4. Full images of western blots.

Figure S5. FACS gating strategies corresponding to Figure 1.

Table S5. List of antibodies used in this study.

Supplementary Data S1. AP-MS dataset.

Supplementary Data S2. BioID dataset.

References

Case descriptions

Patient 1, II.2

Patient is a 32-year-old non-consanguineous Finnish woman with atopic eczema since childhood. Her mother (I.2) suffered from psoriasis and a rapidly progressing neurodegenerative disease that was classified as Amyotrophic Lateral Sclerosis (ALS). She passed away 4 months after initial symptoms (leg pain). Her stepsister (II.3) had psoriasis and pectus excavatum.

Regarding infections, the patient's childhood was unremarkable. After age 12y her growth stunted and she developed recurrent respiratory infections that continued throughout her adult life (mainly rhinosinusitis). Her respiratory illnesses always lasted for at least 3 weeks. After age 15, she has had three episodes of pneumonia. On average she has received 5 to 7 courses of antibiotics annually.

At age of 16 she developed oligoarthritis affecting knees and wrists. Diagnosis of erosive seronegative rheumatoid arthritis was made, and antirheumatoid medication was prescribed with hydroxychloroquine and cortisone injections. A year later, hypogammaglobulinemia was noted with IgG of 3,0 g/l, IgA 0,15 g/l, and IgM 0,17 g/l. At age 17, intravenous immunoglobulin was initiated but treatment was discontinued after three infusions due to an undefined skin reaction. Her arthritis remained active, and methotrexate was added to her medication. Patient delivered a baby in 2007. During pregnancy, antirheumatoid medication had been discontinued. After delivery, her arthritis flared again and methotrexate was recommenced. She received a wrist joint replacement in 2008, after which her recovery was uneventful. Her joints became symptomless, and patient discontinued methotrexate in 2013. At the age of 30, her immunoglobulin levels were checked again (Table S2). Response to tetanus vaccine was suboptimal, 0,03 IU/ml to 0,08 IU/ml. She did not respond to all tested 10 pneumococcal polysaccharide serotypes (1, 4, 5, 6B, 7F, 9V, 14, 18C, 19F, 23F) (15). Subcutaneous immunoglobulin therapy was reinitiated and continues to this day.

She has suffered from chronic diarrhea since age 31. Gastroscopy, colonoscopy, and magnetic resonance imaging of the small intestine revealed no pathologic lesions. Biopsies were normal. Fecal samples were negative for pathogenic bacteria and viruses, including enterovirus. At age 31.6, she had a fever and rash consistent with *pityriasis rosea*. Respiratory samples were positive for picornavirus while stool culture was negative for enterovirus. However, recently her fecal samples have become repeatedly positive for adenovirus and her diarrhea continues.

At age 32, patient was admitted to the hospital with respiratory symptoms and high fever. Chest X-rays were normal. C-reactive protein was 149 mg/l. Moxifloxacin therapy was prescribed, and fever and her respiratory symptoms resolved. However, chronic EBV viremia was noted, fluctuating between 100-26000 viral copies/ml. No CMV or HHV6 viremia was detected.

Her arthritis has flared, and oral methylprednisolone 5 mg daily was prescribed by rheumatologist. In the most recent follow-up, patient is feeling unwell. She has lost 8 kg of weight. EBV viremia is still at low level (350 viral copies/ml). No lymphadenopathy has been noted. She is currently receiving subcutaneous immunoglobulin, methotrexate, and peroral prednisolone.

Patient 2, III.1

Patient 2 is the son of the index case, born from uneventful pregnancy by a Cesarean section at week 41+3 (3865g/52cm/36cm/Apgar 10). There was no history of miscarriages. For the first months of life he was breastfed and developed normally. Intrauterine and early postnatal growth (0 SD) were normal, but growth has since deteriorated into -4 SD and -5 SD range (Figure S1). At age 6 months, he presented

with postprandial vomiting, loose stools, and failure to thrive tachypnea and with gas exchange defect requiring respiratory support with continuous positive airway pressure. High-resolution computer tomography of the lungs (HRCT) showed alveolar and ground glass changes. Bronchoalveolar lavage recovered macrophages (80%; lymphocytes 12%, neutrophils 8%) *Pneumocystis jirovecii*. Mycoplasmas, other bacteria, viruses (CMV) and fungi were excluded by cultures, staining and PCR. Lung biopsy histology was consistent with chronic pneumonitis of infancy. NEHI (neuroendocrine hyperplasia of infancy) was excluded by negative Bombesin staining. No fibrosis, alveolar proteinosis nor vasculitis was found. There was Type 2 pneumocyte hyperplasia, and the expression of surfactant proteins A, B, C, and D was normal. Despite increased macrophages in bronchoalveolar lavage, no alveolar macrophage infiltration was found. Prednisolone was started with good response.

At 3 years HRCT had normalized and patient showed respiratory distress only during infections; the symptoms were controlled by inhaled budesonide and salbutamol. At age 6, the patient developed *Moraxella catarrhalis* pneumonia. HRCT again showed ground glass changes, as well as bronchiectasis and calcified perihilar lymph nodes.

From 6 months onwards, the patient also displayed gastrointestinal symptoms such as postprandial vomiting and discomfort, loose and occasionally bloody stools, and poor appetite. Gastroscopy and ileocolonoscopy at 18 months of age showed macroscopically normal intestinal mucosa; colonic biopsies showed edematous lamina propria and absent lymphoid follicles. Control endoscopies at age 3 and 4 years showed duodenal villous atrophy with intraepithelial lymphocytosis, large transformed lymphocytes, and mild eosinophilia. Aphthous changes, lymphoid hyperplasia as well as neutrophilic cryptitis and crypt abscesses with loss of Paneth cells were observed in terminal ileum. Gastrostomy feeding, elimination diets (cow milk, gluten) and peroral prednisolone were started at age 2 but did not improve the situation. At age 4, peroral tacrolimus and parenteral nutrition resolved gastrointestinal inflammation and improved his growth. The patient is positive for anti-Goblet cell autoantibodies. No other enterocyte autoantibodies have been detected.

When 24 months old, the patient developed chronic perioral and perianal eczema. At age 5, the eczema flared into psoriasiform skin irritation of the trunk, scalp, palms and soles. Age 8, he developed erythroderma and disabling rhagades on palms and soles (Figure S1). Skin biopsy confirmed psoriasis vulgaris, but was non-responsive to peroral tacrolimus, acitretin or PUVA photochemotherapy. When 8 years, subcutaneous methotrexate was started with partial response.

The patient was first noted to have hypogammaglobulinemia (P-IgG 0.7 g/l) at age 18 months, but plasma immunoglobulin levels transiently recovered when gut inflammation was controlled. These decreased again at age 3 to IgG 0.9g/l, IgA 0.18 g/l, IgM 0.25g/l, IgE 19 kU/l. He responded to only one of 10 tested pneumococcal serotypes (1, 4, 5, 6B, 7F, 9V, 14, 18C, 19F, 23F) while tetanus antibody response was normal [11]. Immunoglobulin replacement therapy was initiated at age 3 and has continued ever since. During the course of the study, the patient has received peroral prednisolone, tacrolimus and subcutaneous methotrexate.

In January 2017, after conditioning with fludarabine, treosulfan and thiotepa, he underwent matched (10/10) unrelated allogeneic hematopoietic stem cell transplant (HSCT). He developed hypertensive crisis responsive to medication. After HSCT, hematopoietic recovery was rapid, skin and GI tract normalized without signs of graft versus host disease. In follow-up, increased chimerism with host-derived cells (1.9-31-13%, of CD3+ cells up to 45%) has led to the tapering of immunosuppressive medication. Three months after HSCT, he developed respiratory distress. Bronchoalveolar lavage, showed *Actinomyces viscosus* and lung biopsies showed patchy alveolar wall fibrosis, possibly as an end

result of interstitial pneumonia. Focal fibrosis in walls of arteries following bronchioles but not in smaller peripheral arterioles was noted. Due to subpleural myofibroblast proliferation, pulmonary hypertension was suspected. Within 6 months he rejected the allograft and the disease returned. One year after the transplant he died for progressive pulmonary fibrosis.

Table S1. Genes affecting vesicular transport and causing monogenic autoimmune disease [12-17].

**Gene**	LRBA	COPA	ITCH	TOM1	AP1S3
**Gene function**	Regulator of endosomal trafficking	Subunit of COPI carrier complex	E3 ubiquitin ligase		Component of adaptor protein complex 1
**Inheritance**	AR	AD	AR	AD	AD
**Number of described cases**	>40	21	10	2	15
**Main autoimmune features**	Enteropathy, cytopenias, interstitial lung disease	Arthritis, interstitial lung disease	Lung disease, liver disease, endocrinopathy, enteropathy	Enteropathy, psoriasis, interstitial lung disease	Pustular psoriasis
**Immunodeficiency**	Recurrent respiratory tract infections	-	-	Recurrent respiratory tract infections, EBV-viremia	-
**Other features**	Lymphoproliferation & splenomegaly	-	Developmental delay, dysmorphic features	-	-
**Lab alterations**	Regulatory T cell deficiency, reduced switched memory B cells, antibody deficiency	Increased Th17 cells	NA	Regulatory T cell deficiency, reduced switched memory B cells, antibody deficiency	-
**Proposed pathogenic mechanism**	Defective recycling of CTLA4 receptors and loss of CTLA4 surface expression; defective autophagy	Increased ER stress, defective autophagy	NA	Impaired recruitment to endosomes; defective autophagy	Impaired TLR3 recycling and interferon production

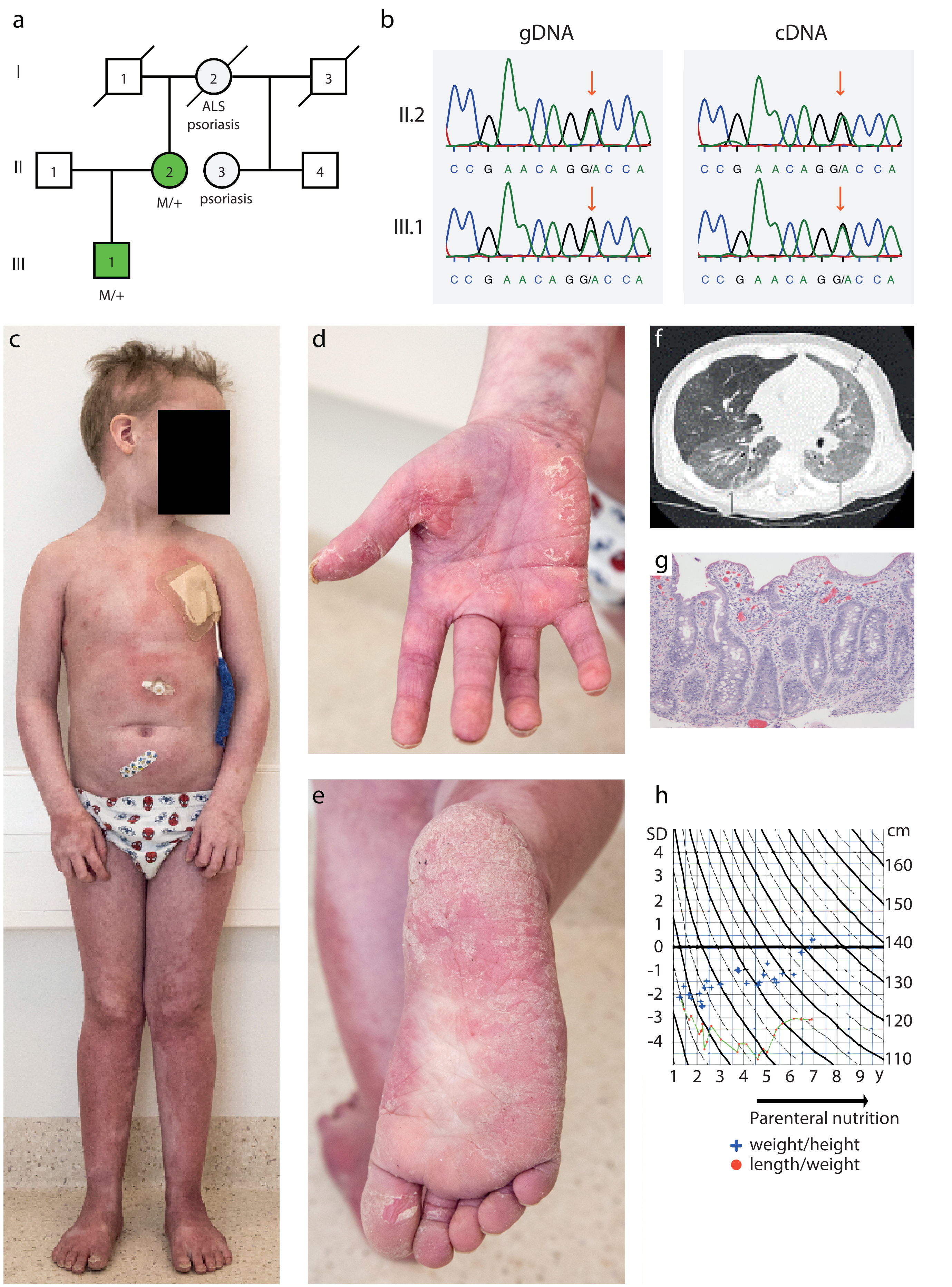

Figure S1: Clinical and genetic findings. **a**) Family pedigree. Individuals I.1, I.2, I.3, II.3, II.4 were unavailable for sampling or clinical examination.
b) Sanger sequencing trace of DNA and RNA/cDNA isolated from primary patient fibroblasts. The TOM1 mutation is indicated with red arrow.
c) Patient III.1: disseminated psoriasis vulgaris, with large lesions in both legs and around PEG and Hickman line inlets. The lesions in the scalp led to bald patches. The patient also has rhagades in palms (**d**) and soles (**e**).
f) Lung HR-CT at age of 7 months with ground glass changes and thickening of interlobular septae.
g) Duodenal biopsy showed villous atrophy with intraepithelial lymphocytosis, large transformed lymphocytes, and mild eosinophilia.
h) Growth curve, showing stunted growth between -3 and -5SD.

Table S2. Immunological features of study participants.

Patient	Reference range	Female II.2<br>32y		Male, III.1<br>2-3y / 9y
**Leukocytes**	3400-8200	3500		25600 / 10600
**Lymphocytes**	1300-3600<br>1700-6900/1100-5900 *	600 ↓		7000 (inc.) / 1670
**Monocytes**	200-800	300		600 / 550
**Neutrophils**	1500-6700	2300		9930 / 8210
**Basophils**	0-100	10		20 / 10
**Eosinophils**	30-440	10		5010 (inc.) / 110
**Platelets**	150 000-360 000	173 000		422 000 / 296 000
**B-cells (CD19+)**	100-500<br>200-2100/ 200-1600 *	10 ↓		970 / 320
**T cells (CD3+)**	900-4500/700-4200*	470 ↓		4390 / 1040
**CD3+CD4+**	300-1400<br>500-2400/ 300-2000 *	370 ↓		3006 / 722
**CD3+CD8+**	200-1200<br>300-1600/00-1800 *	130 ↓		1250 / 300
**NK-cells ($CD3^-CD16^+56^+$)**	90-600<br>100-1000/ 90-900 *	60 ↓		1360 (inc.) / 20 ↓
**NK cell function and degranulation**		normal		normal
**Dendritic cells**				
**Plasmacytoid ($lin^-HLA\text{-}DR^+CD123^+CD11c^-$)**	0.1-0.3%	0.04 ↓		0.04 ↓
**Monocytoid ($lin^-HLA\text{-}DR^+CD123^-CD11c^+$)**	0.1-0.3%	0.08 ↓		0.07 ↓
**Immunoglobulins**				
**IgG**	6.8-15.0 g/L	0.9 ↓		0,8 ↓ / 7,5 (subst.)
**IgA**	0.52-4.02 g/L	0 ↓		0,25 / <0.1 ↓
**IgM**	0.47-2.84 g/L	0.07 ↓		0,62 / 0.1 ↓
**IgE**	0-110 IU/L	<4		19 kU/L / 5 kU/L
**Functional studies**				
**Complement**	Classical, alternative, mannan-binding lectin pathway hemolytic activities (%)	CH50 108<br>AH50 91<br>LH50 0		CH100Cl 87 % (N)<br>CH100Al 52% (N)<br>CH100L 37% (N)
**Lymphocyte proliferative responses to mitogens**	Phytohemagglutinin, concanavalin A, pokeweed mitogen	Normal		PHA (N)<br>ConA↓ (CD456%,CD8 63%)<br>PWM (N)
**Specific antibodies to tetanus (before/4 weeks after)**	> 0.1 SPU/ml	0.03	0.08 ↓	0,12 / 1.1 (subst.)
**Specific antibody titers against polysaccharide vaccine antigens (Pneumovax;before/4 weeks after)**	>0.35 ug/ml	1/10 detectable	0/10 detectable ↓	1/10 responded ↓

*Reference values/range for children 2-5yrs/ 5-10yrs 5. -95. percentile for age

Table S3. Lymphocyte differentials of study participants.

	Cell type	Control Range (%)	Female II.2 32 y	Male, III.1 7 y / 9y
**$CD19^{+}$ B cells**		5-22	2	22 / 24
**Transitional**	$CD38^{hi}IgM^{hi}$	0.6-3.5	0	5.8 / 0.5
**Naive**	$CD27^{-}IgD^{+}$	43,2-82,4	80.1	96.6
**Memory**	$CD27^{+}$	15-45	16.7	2.0 ↓ / 2.2 ↓
**Marginal zone-like**	$CD27^{+}IgD^{+}IgM^{+}$	7.2-30.8	11.4	2.0↓ / 1.6 ↓
**Switched memory**	$CD27^{+}IgD^{-}IgM^{-}$	6.5-29.2	0 ↓	0.0↓ / 0.4 ↓
**Plasmablasts**	$CD38^{++}IgM^{-}$	—	0	<0.2%
**Activated**	$CD38^{low}CD21^{low}$	0.6-3.5	18.4 ↑	0 / ? 100% CD19+CD21 low
**CD3+ T cells**		65.9–87.6	92	74
	CD4-CD8-TCRab+	0,3-3,3	2.0	1.9
	TCRgd+	1,9-11,7	0.6	1.7
**CD3+CD4+ T cells**		35.6–56.0	66.9 ↑	50.1
**Naïve**	$CCR7^{+}CD45RA^{+}$	20.5-54.8	65.1 ↑	79.7
**TCM**	$CCR7^{+}CD45RA^{-}$	8.4-32.8	31.6	17.5
**TEM**	$CCR7^{-}CD45RA^{-}$	19.9-52.4	3.0 ↓	2.7 ↓
**TEMRA**	$CCR7^{-}CD45RA^{+}$	1.4-17.0	0.3 ↓	0.1 ↓
**Activated**	$HLADR^{+}CD38^{-}$	2.4-9.6	0.9 ↓	1.0
	$HLADR^{-}CD38^{+}$	40.4-72.9	81.7 ↑	79.4
	$HLADR^{+}CD38^{+}$	0.9-4.6	1.6	0.4
**RTE**	$CD45RA^{+}CD62L^{+}CD31^{+}$	14.4-38.3	41.2 ↑	45.2
**Th17**	$CD45RA^{-}CD4^{+}CCR6^{+}CXCR3^{-}$	19-34	16.4	18.7
**Th1**	$CD45RA^{-}CD4^{+}CCR6^{-}CXCR3^{+}$	16-32	46.2	18
**Th1/Th17**	$CD45RA^{-}CD4^{+}CCR6^{+}CXCR3^{+}$	27-40	24.5	9.5
**IFNγ production (PMA stimulation)**		11-16	6.3	0.3
**IL-17 production $CD4^{+}CD69^{+}$ cells**		0.7-1.7	0.37	0.14
**CD3+CD8+ T-cells**		13.1–34.5	24	21
**Naive**	$CCR7^{+}CD45RA^{+}$	18.8-71.0	87.3 ↑	94.2
**TCM**	$CCR7^{+}CD45RA^{-}$	1.2-7.3	6.6	1.7
**TEM**	$CCR7^{-}CD45RA^{-}$	14.6-63	3.7 ↓	3.5 ↓
**Temra**	$CCR7^{-}CD45RA^{+}$	4.5-33.7	2.4 ↓	0.6 ↓
**Activated**	$HLADR^{+}CD38^{-}$	3.8-32.4	2.7	1.1
	$HLADR^{-}CD38^{+}$	30.3-78.5	72.2	62.8
	$HLADR^{+}CD38^{+}$	1.4-21.4	6.2	0.4
**Treg**	$CD25^{hi}CD127^{lo}$	2.8-6.4	5.8	5.5
**Treg suppressive capacity**		normal	normal	poor

Table S4. List of shared coding variants between the patients. Autosomal dominant high-confidence variants present in both affected individuals that affect the coding sequence. Variants localizing to polymorphic genes or variants present in healthy control datasets (The Exome Aggregation Consortium (ExAC), 1000 Genomes, NHLBI Exome variant server, and UK TWIN and ALSPAC study cohorts [18-20] as well as in-house databases) have been excluded.

chr	position	Allele	Gene	ENS	Transcript	Amino acid change	rs #	SIFT	PolyPhen
1	84610142	A/C	PRKACB	ENSG00000142875	ENST00000370685	H33P	-	tolerated	benign
12	54379208	G/C	HOXC10	ENSG00000180818	ENST00000303460	K55N	-	deleterious	possibly_damaging
22	35728994	G/A	TOM1	ENSG00000100284	ENST00000411850	G307D	-	deleterious	probably_damaging
3	51423687	A/G	MANF	ENSG00000145050	ENST00000528157	D48G	-	tolerated	benign
8	124109674	C/A	TBC1D31	ENSG00000156787	ENST00000287380	S275Y	-	deleterious	possibly_damaging
2	29287927	INS->TGC	C2orf71	ENSG00000179270	ENST00000331664	Inframe p.1225	-	-	-
5	140953564	INS->GAGGAG	DIAPH1	ENSG00000131504	ENST00000398557	Inframe p.618	-	-	-
9	12775862	INS->GGCGGCGGC	LURAP1L	ENSG00000153714	ENST00000319264	Inframe p.50	-	-	-
14	74205942	INS->TGCTGCTGT	ELMSAN1	ENSG00000156030	ENST00000286523	Inframe p.257	-	-	-
14	101350671	INS->CTT	RTL1	ENSG00000254656	ENST00000534062	Inframe p.152	-	-	-
16	67229794	INS->GCA	E2F4	ENSG00000205250	ENST00000379378	Inframe p.306	-	-	-
18	47405426	INS->GAG	MYO5B	ENSG00000167306	ENST00000285039	Inframe p.1055	-	-	-
18	51750585	INS->CGC	MBD2	ENSG00000134046	ENST00000256429	Inframe p.115	-	-	-

Supplementary Data S1. AP-MS dataset.
The high-confidence protein-protein interactions for TOM1, TOM1-G307D, and TOLLIP, identified using AP-MS. The abundance of each interactor was defined using spectral counting.

Supplementary Data S2. BioID dataset.
The high-confidence protein-protein interactions for LRBA, TOLLIP and TOM1, identified using BioID-MS. The abundance of each interactor was defined using spectral counting.

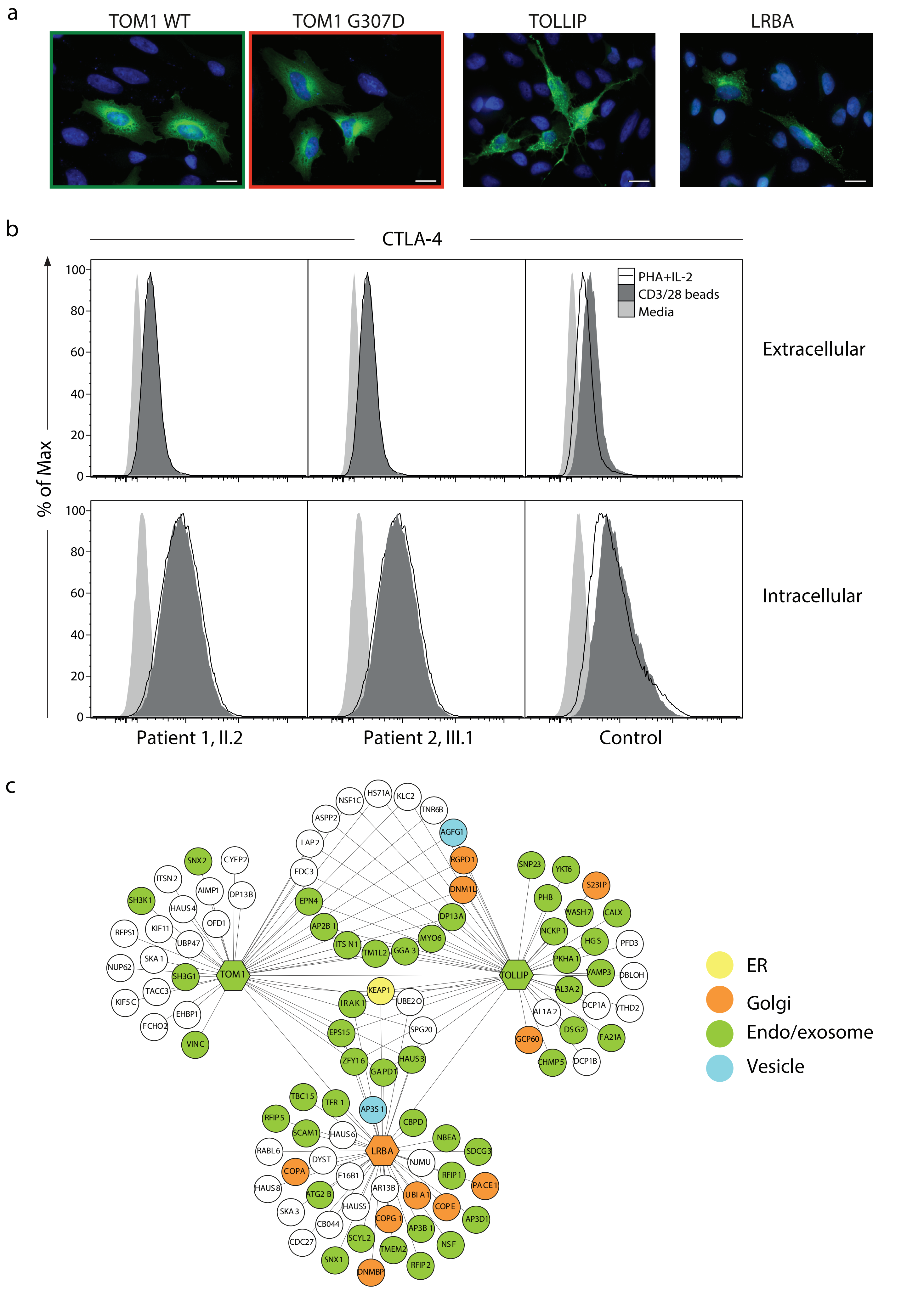

Figure S2: Differential molecular context of TOM1 and LRBA and role in CTLA-4 regulation.
a) Immunofluorescence microscopy shows differential localization of TOM1 WT and mutant compared to TOLLIP and LRBA.
b) The patient cells do not indicate a defect in CTLA-4 expression. **c**) TOM1 shares many interactions with TOLLIP and some with LRBA.

a

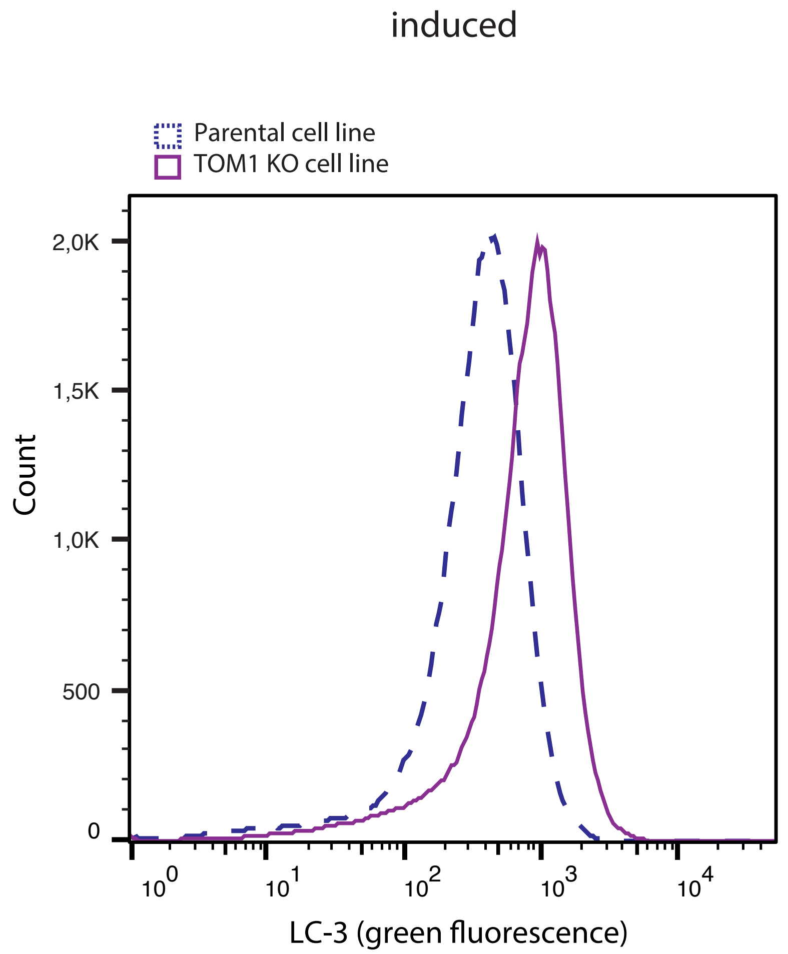

b

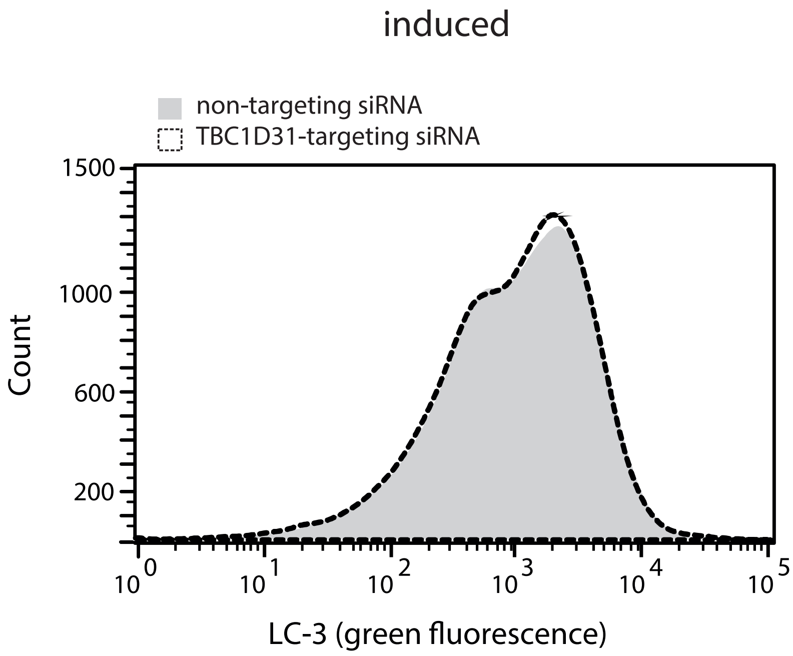

Figure S3: Induction of autophagy in TOM1 knock-out HAP1 cells and in TBC1D31 siRNA treated parental HAP1 cells.
a) TOM1 KO cell lines responds to autophagy induction as visualized by increased LC-3 staining compared to parental cell line.
b) Knock-down of TBC1D31 does not affect the cells response to autophagy induction,
as LC-3 staining of non-targeting siRNA treated cells is overlapping with TBC1D31 siRNA treated cells.

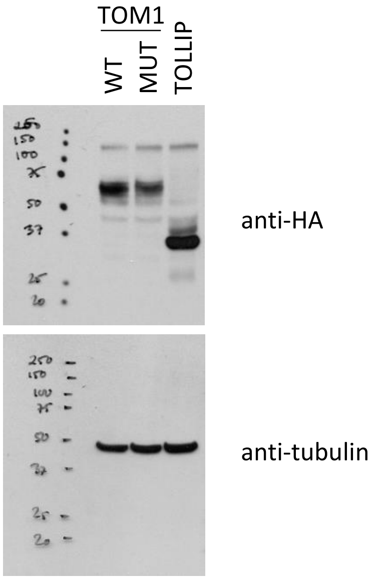

Figure S4: Full, un-cropped images of blots.

a

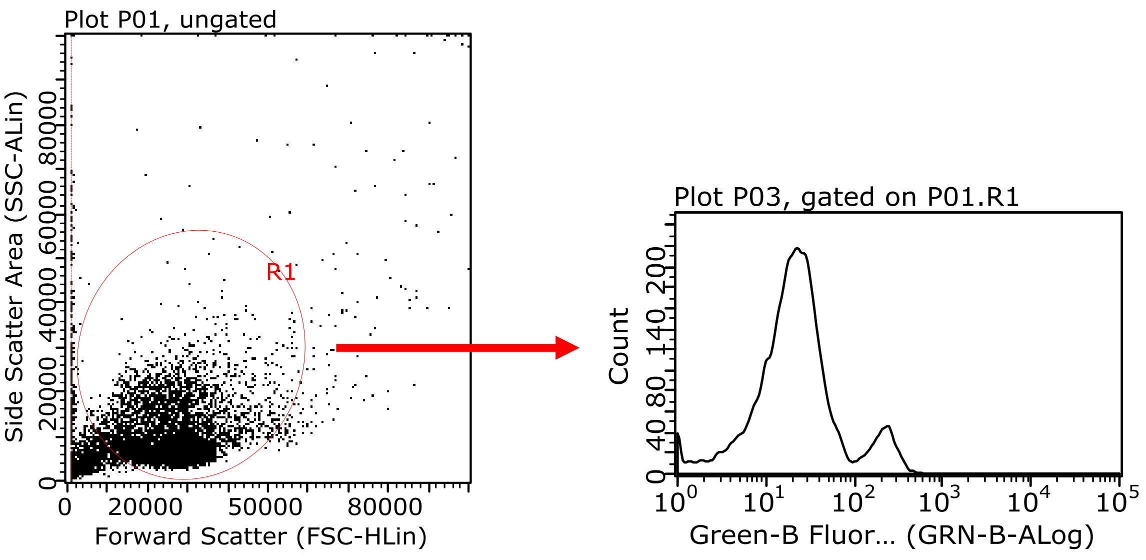

b

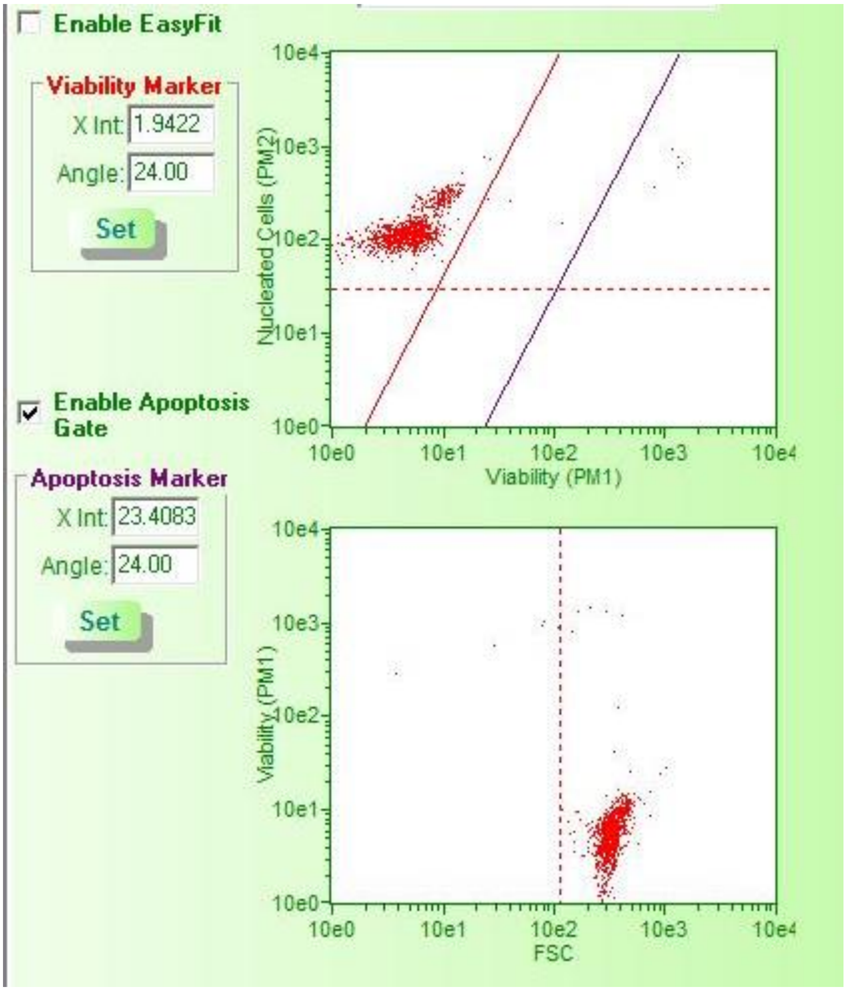

Figure S5: FACS gating strategies for (**a**) LCIII-staining corresponding to FIG1D-E, and (**b**) for staining of viable, apoptotic and dead cells corresponding to FIG1F.

Table S5. List of antibodies used in this study.

Antibody	Catalog Number	Company	Used dilution
Mouse monoclonal anti-HA.11	Cat# 901514	BioLegend	1:3000
Mouse monoclonal (DM1A) to alpha Tubulin	AB7291	Abcam	1:8000
ECL mouse IgG, HRP-linked whole Ab (from sheep)	NA931	GE Healthcare	1:4000
Anti-LC3 FITC, clone 4E12	Part No. CS208214	Merck	1:20
ViaCount Reagent	4000-0040	Merck	380 µl + 20 µl sample
Maxpar Signaling I Panel Kit, 7 markers	201309	Fluidigm	each antibody 1:100
Mouse monoclonal anti-V5	R960-25	Invitrogen	
Alexa-488 goat anti-mouse IgG	A-11001	Thermo Fisher Scientific	1:300
Alexa-594 goat anti-rabbit IgG	A-11012	Thermo Fisher Scientific	1:300
Rabbit polyclonal to GRP78 BiP	AB21685	Abcam	1:200

References

1 Ilander, M. *et al.* Enlarged memory T-cell pool and enhanced Th1-type responses in chronic myeloid leukemia patients who have successfully discontinued IFN-alpha monotherapy. *PLoS One* **9**, e87794, doi:10.1371/journal.pone.0087794 (2014).

2 Haapaniemi, E. M. *et al.* Autoimmunity, hypogammaglobulinemia, lymphoproliferation, and mycobacterial disease in patients with activating mutations in STAT3. *Blood* **125**, 639-648, doi:10.1182/blood-2014-04-570101 (2015).

3 Chiang, S. C. *et al.* Comparison of primary human cytotoxic T-cell and natural killer cell responses reveal similar molecular requirements for lytic granule exocytosis but differences in cytokine production. *Blood* **121**, 1345-1356, doi:10.1182/blood-2012-07-442558 (2013).

4 Ma, C. S. *et al.* Monogenic mutations differentially affect the quantity and quality of T follicular helper cells in patients with human primary immunodeficiencies. *J Allergy Clin Immunol* **136**, 993-1006 e1001, doi:10.1016/j.jaci.2015.05.036 (2015).

5 Ruitenberg, J. J., Boyce, C., Hingorani, R., Putnam, A. & Ghanekar, S. A. Rapid assessment of in vitro expanded human regulatory T cell function. *J Immunol Methods* **372**, 95-106, doi:10.1016/j.jim.2011.07.001 (2011).

6 Varjosalo, M. *et al.* The protein interaction landscape of the human CMGC kinase group. *Cell Rep* **3**, 1306-1320, doi:10.1016/j.celrep.2013.03.027 (2013).

7 Heikkinen, T. *et al.* Somatic MED12 Nonsense Mutation Escapes mRNA Decay and Reveals a Motif Required for Nuclear Entry. *Hum Mutat* **38**, 269-274, doi:10.1002/humu.23157 (2017).

8 Varjosalo, M. *et al.* Application of active and kinase-deficient kinome collection for identification of kinases regulating hedgehog signaling. *Cell* **133**, 537-548, doi:10.1016/j.cell.2008.02.047 (2008).

9 Turunen, M. *et al.* Uterine leiomyoma-linked MED12 mutations disrupt mediator-associated CDK activity. *Cell Rep* **7**, 654-660, doi:10.1016/j.celrep.2014.03.047 (2014).

10 Huang da, W., Sherman, B. T. & Lempicki, R. A. Systematic and integrative analysis of large gene lists using DAVID bioinformatics resources. *Nat Protoc* **4**, 44-57, doi:10.1038/nprot.2008.211 (2009).

11 Timby, N. *et al.* Infections in infants fed formula supplemented with bovine milk fat globule membranes. *J Pediatr Gastroenterol Nutr* **60**, 384-389, doi:10.1097/MPG.0000000000000624 (2015).

12 Alkhairy, O. K. *et al.* Spectrum of Phenotypes Associated with Mutations in LRBA. *J Clin Immunol* **36**, 33-45, doi:10.1007/s10875-015-0224-7 (2016).

13 Gamez-Diaz, L. *et al.* The extended phenotype of LPS-responsive beige-like anchor protein (LRBA) deficiency. *J Allergy Clin Immunol* **137**, 223-230, doi:10.1016/j.jaci.2015.09.025 (2016).

14 Lo, B. *et al.* AUTOIMMUNE DISEASE. Patients with LRBA deficiency show CTLA4 loss and immune dysregulation responsive to abatacept therapy. *Science* **349**, 436-440, doi:10.1126/science.aaa1663 (2015).

15 Lohr, N. J. *et al.* Human ITCH E3 ubiquitin ligase deficiency causes syndromic multisystem autoimmune disease. *Am J Hum Genet* **86**, 447-453, doi:10.1016/j.ajhg.2010.01.028 (2010).

16 Setta-Kaffetzi, N. *et al.* AP1S3 mutations are associated with pustular psoriasis and impaired Toll-like receptor 3 trafficking. *Am J Hum Genet* **94**, 790-797, doi:10.1016/j.ajhg.2014.04.005 (2014).

17 Watkin, L. B. *et al.* COPA mutations impair ER-Golgi transport and cause hereditary autoimmune-mediated lung disease and arthritis. *Nat Genet* **47**, 654-660, doi:10.1038/ng.3279 (2015).
18 Auer, P. L. *et al.* Imputation of exome sequence variants into population- based samples and blood-cell-trait-associated loci in African Americans: NHLBI GO Exome Sequencing Project. *Am J Hum Genet* **91**, 794-808, doi:10.1016/j.ajhg.2012.08.031 (2012).
19 Consortium, U. K. *et al.* The UK10K project identifies rare variants in health and disease. *Nature* **526**, 82-90, doi:10.1038/nature14962 (2015).
20 Genomes Project, C. *et al.* An integrated map of genetic variation from 1,092 human genomes. *Nature* **491**, 56-65, doi:10.1038/nature11632 (2012).
